# A prognostic signature based on cuprotosis-related long non-coding RNAs predicts the prognosis and sensitivity to chemotherapy in patients with colorectal cancer

**DOI:** 10.3389/fmed.2022.1055785

**Published:** 2022-11-16

**Authors:** Wei Li, Guiyun Yang, Hao Dong, Jiajing Zhu, Tongjun Liu

**Affiliations:** ^1^Department of Colorectal and Anal Surgery, The Second Hospital of Jilin University, Changchun, China; ^2^Department of Operating Room, The Second Hospital of Jilin University, Changchun, China; ^3^Department of Gastrointestinal Nutrition and Hernia Surgery, The Second Hospital of Jilin University, Changchun, China; ^4^Department of Radiology, China-Japan Union Hospital of Jilin University, Changchun, China

**Keywords:** colorectal cancer, cuprotosis, prognostic signature, immune infiltration, immunotherapy, chemotherapy sensitivity

## Abstract

Cuprotosis, a newly proposed mechanism of cell death, can trigger acute oxidative stress that leads to cell death by mediating protein lipidation in the tricarboxylic acid cycle. However, cuprotosis-related long non-coding RNAs (CRLNCs) and their relationship with prognosis and the immunological landscape of colorectal cancer (CRC) are unclear. We have developed a lncRNA signature to predict survival time, immune infiltration, and sensitivity to chemotherapy. CRLNCs were screened using the Cor function of the R software and the differentially expressed lncRNAs were collected with the limma package. Differentially expressed long non-coding RNAs (lncRNAs) associated with prognosis were selected using univariate regression analysis. A prognostic signature was developed using the least absolute shrinkage and selection operator (LASSO) and multivariate regression analysis. Patients with CRC were divided into two groups based on the risk score. The low-risk group had a more favorable prognosis, higher expression of immune checkpoints, and a higher level of immune cell infiltration compared with the high-risk group. Furthermore, there was a close association between the risk score and the clinical stage, tumor mutational burden, cancer stem cell index, and microsatellite instability. We also assessed chemotherapy response in the two risk groups. Our study analyzed the role of CRLNCs in CRC and provided novel targets and strategies for CRC chemotherapy and immunotherapy.

## Introduction

Colorectal cancer (CRC) is responsible for approximately 10% of cancer cases and related deaths worldwide (1). Only in developed countries does the incidence of CRC show a stable or declining trend, which is primarily due to the widespread use of large-scale screening and colonoscopy, as well as the continuous improvement of people’s living and eating habits. It is estimated that there will be 25 million new cases of CRC worldwide by 2035 (2). In addition to surgery, radiotherapy and chemotherapy are still widely applied to reduce recurrence and improve survival. Chemotherapy, which involves the application of chemical compounds to inhibit the growth of tumor cells, is an indispensable part of the treatment process. Currently, platinum-based chemotherapy in combination with 5-fluorouracil is the first-line treatment option in treating patients with CRC (3, 4). However, different patients respond differently to the same chemotherapy regimen, leading to large variations in patient prognoses (5, 6). Exploring new, specific, and effective targets related to chemotherapy sensitivity, as well as recognizing individualized and precise treatment, is therefore critical for CRC therapy.

Copper homeostasis is an ancient phenomenon in living organisms. Copper is an indispensable trace element for the homeostasis of the internal environment (7). Copper contributes to the progression of tumors, such as breast and lung cancer, where it is involved in tumor angiogenesis, epithelial-mesenchymal transition, and cell proliferation and metastasis (8, 9). Therefore, copper-chelating agents have been studied and reported to inhibit tumor growth in some clinical trials (10). Meanwhile, copper can also promote oxidative stress to mediate cell death (11, 12). Copper-specific ionophores can transport copper into cells at specific sites, increasing the copper level in tumor cells, and then mediating the toxicity of copper overload, which results in cell death (10). The role of copper in the treatment of tumors is complex and versatile. Mutations in lncRNAs are believed to mediate several forms of tumor development along with protein-coding genes (13).

LncRNAs can regulate immune and inflammatory responses at the transcriptional and posttranscriptional levels by interacting with proteins, RNA, and DNA (14). At the same time, lncRNAs have a close relationship with the tumor microenvironment (TME) (15). Several lncRNAs, including TUG1, MALAT1, H19, GAS5, LINC00152, UCA1, CUDR, and AA174084, have been identified as predictive biomarkers of CRC. Investigating such lncRNAs as potential targets for CRC therapy is of long-term value. GAS5 is involved in regulating chemotherapy resistance in CRC. The other lncRNAs require further investigation. To determine whether cuprotosis-related long non-coding RNAs (CRLNCs) play a role in CRC, a prognostic signature of the immune infiltration and survival of patients with CRC was developed. A different prognosis was revealed by the Kaplan-Meier analysis. Various methodologies, such as XCELL, TIMER, and ssGSEA, were also used to analyze the immune infiltration in patients with CRC. The analyses of immune checkpoints, clinicopathological data, tumor mutational burden (TMB), cancer stem cells (CSCs), microsatellite instability (MSI), and chemotherapy response were also performed.

## Materials and methods

### Datasets and samples

The transcriptome, mutation, and clinical data for COAD containing 32 healthy tissues and 375 tumors were downloaded from The Cancer Genome Atlas (TCGA) database. Six fresh frozen CRC and paracancerous paired tissues were obtained from the Second Hospital of Jilin University. The cuprotosis-related genes (CRGs) are shown in Supplementary Table 1.

### Identification of differentially expressed cuprotosis-related long non-coding RNAs

To identify lncRNAs closely related to CRGs, we performed a screen using the Cor function of the R software, with the filter conditions set to require a correlation coefficient of >0.3 with a false discovery rate of <0.001. Subsequently, differentially expressed CRLNCs between the 32 normal and 375 tumor samples were selected using the limma package (| log Foldchange| > 1 and false discovery rate < 0.05).

### Construction and validation of a prognostic long non-coding RNA signature

The Supplementary material provide details about the construction and validation of the prognostic lncRNA signature.

### Gene set enrichment analysis and nomogram construction

The gene set enrichment analysis (GSEA) and nomogram are presented in the Supplementary material.

### Immune landscape, immune checkpoints, and clinical data analyses

Analyses of the immune landscape, immune checkpoints, and clinical data are presented in the Supplementary material.

### Analyses of tumor mutational burden, cancer stem cells, and microsatellite instability

Tumor mutational burden is an essential marker of immunotherapy response and prognosis. Therefore, we compared genetic mutations in samples from low-risk and high-risk groups. The mutational burdens from all samples were then calculated and compared. A correlation analysis was applied to determine the significant relationships between the risk scores, TMB, and immune infiltration. We also explored the link between CRGs and risk scores. MSI could reflect the effect of immunotherapy. Therefore, the association between MSI and risk score was analyzed. We compared patients’ survival times between MSI-H and MSS/MSI-L. We also integrated MSI into the signature for survival analysis.

### Drug sensitivity analysis and identification of differential genes

The limma package was used to identify the differentially expressed genes (DEGs) (| log Foldchange| > 1 and false discovery rate < 0.05). To further search for the hub genes, we used the CytoNCA plugin in Cytoscape software. Based on the scores of Betweenness, Closeness, Degree, Eigenvector, LAC, and Network, we screened the DEGs twice to obtain core genes. Furthermore, Gene Ontology and Kyoto Encyclopedia Genes and Genomes pathway enrichment analyses were used to explore the functional pathways based on the DEGs. Finally, to investigate the differences in response to chemotherapy, we used the pRRophetic package to predict drug sensitivity.

### Quantitative real-time PCR

Total RNA was extracted from CRC tissues using Trizol reagent (Invitrogen, Carlsbad, CA, United States). We used a reverse transcription kit (Takara, Tokyo, Japan) to synthesize cDNA. The SYBR Premix Ex Taq™ kit (Takara, Japan) was used to perform the quantitative real-time PCR (RT-qPCR). The expression level of LINC00412, AC016737.1, AC026782.2, AC090204.1, AC129507.1, and AC116914.2 was normalized using glyceraldehyde-3-phosphate dehydrogenase. The data were analyzed using the 2^–ΔΔ*Ct*^ method. The primers of the seven genes are listed in Supplementary Table 2.

### Statistical analyses

All statistical analyses were performed using R version 4.1.1. *P* < 0.05 was considered significant.

## Results

### Analysis of differentially expressed cuprotosis-related long non-coding RNAs

**FIGURE 1:**
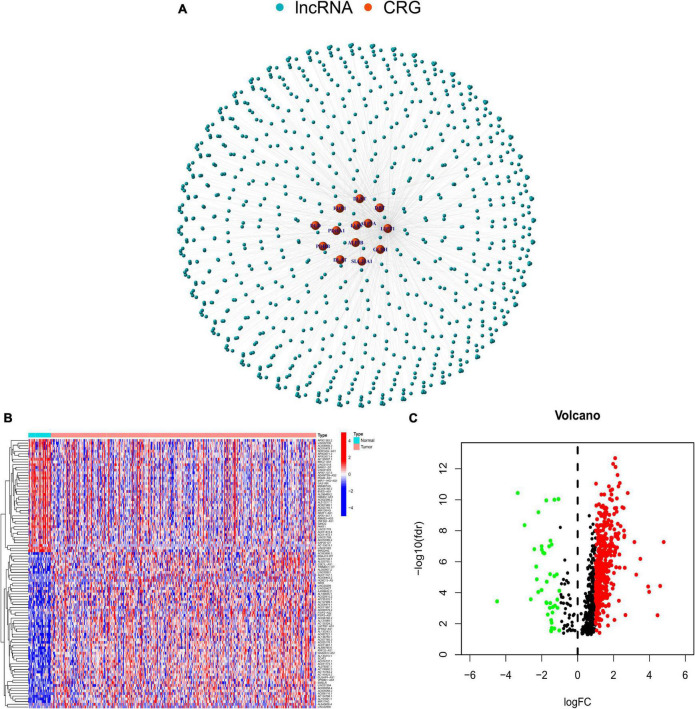
**(A)** Network of long noncoding RNAs and cuprotosis-related genes. **(B)** Differential expression of cuprotosis-related long non-coding RNAs (CRLNCs) between normal and tumor tissues. **(C)** The volcano plot of CRLNCs. Red represents upregulated CRLNCs; green represents downregulated CRLNCs. CRGs, cuprotosis-related genes; CRLNCs, cuprotosis-related long non-coding RNAs.

The study design is presented in Supplementary Figure 1. The Cor function was performed to select 880 CRLNCs. The association between CRGs and lncRNAs is shown in Figure 1A. We discovered 487 CRLNCs that were differentially expressed, with 445 being upregulated in CRC and 42 being downregulated (Figures 1B–C).

### Construction and validation of the prognostic signature

**FIGURE 2:**
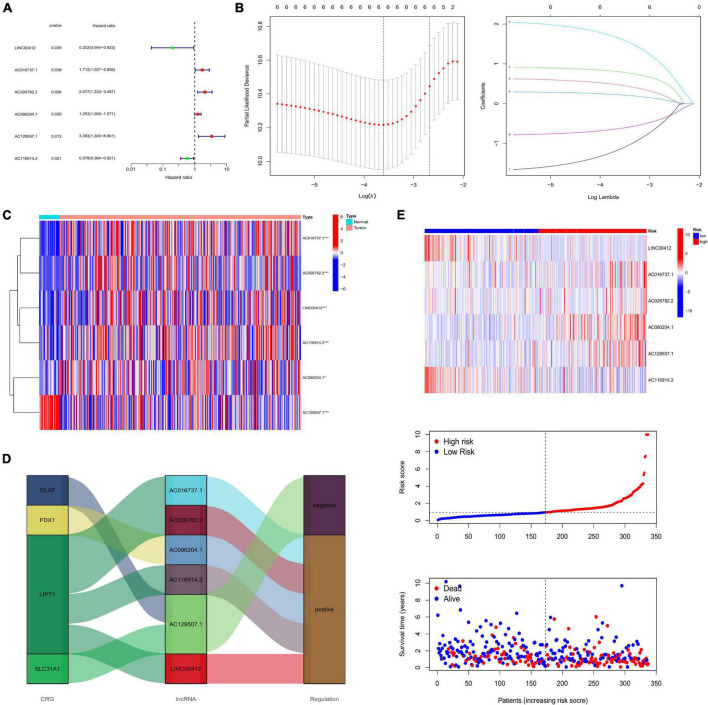
**(A)** Univariate Cox regression analysis. **(B)** The LASSO algorithm further selected the most crucial genes and LASSO coefficient profiles. **(C)** Expression of risk lncRNAs between normal and tumor tissues. **(D)** Sankey diagram showing the relationship between CRGs and lncRNAs. **(E)** Heatmap showing the expression of risk lncRNAs between low- and high-risk groups, and the ranked dot plot showing the risk score distribution in all samples. (*P < 0.05; **P < 0.01; ***P < 0.001; ns, not significant). LASSO, least absolute shrinkage and selection operator; CRGs, cuprotosis-related genes.

As a result of the univariate regression analysis, six CRLNCs were discovered to be linked with the prognosis of patients with CRC (Figure 2A). We then selected the genes corresponding to the smallest lambda value for the multivariate Cox regression analysis (Figures 2B–C). Finally, LINC00412, AC016737.1, AC026782.2, AC090204.1, AC129507.1, and AC116914.2 were screened to construct the risk signature. The formula of the risk signature is as follows:


R⁢i⁢s⁢k⁢s⁢c⁢o⁢r⁢e=(-1.73912912346949⁢e*⁢x⁢p⁢r⁢e⁢s⁢s⁢i⁢o⁢n⁢o⁢f⁢L⁢I⁢N⁢C⁢00412)

+(0.6423027570568⁢e*⁢x⁢p⁢r⁢e⁢s⁢s⁢i⁢o⁢n⁢o⁢f⁢A⁢C⁢016737.1)

+(0.927870667759444⁢e*⁢x⁢p⁢r⁢e⁢s⁢s⁢i⁢o⁢n⁢o⁢f⁢A⁢C⁢026782.2)

+(0.306445754811284⁢e*⁢x⁢p⁢r⁢e⁢s⁢s⁢i⁢o⁢n⁢o⁢f⁢A⁢C⁢090204.1)

+(2.09976593806317⁢e*⁢x⁢p⁢r⁢e⁢s⁢s⁢i⁢o⁢n⁢o⁢f⁢A⁢C⁢129507.1)

+(-0.802467801481654⁢e*⁢x⁢p⁢r⁢e⁢s⁢s⁢i⁢o⁢n⁢o⁢f⁢A⁢C⁢116914.2).

**FIGURE 3:**
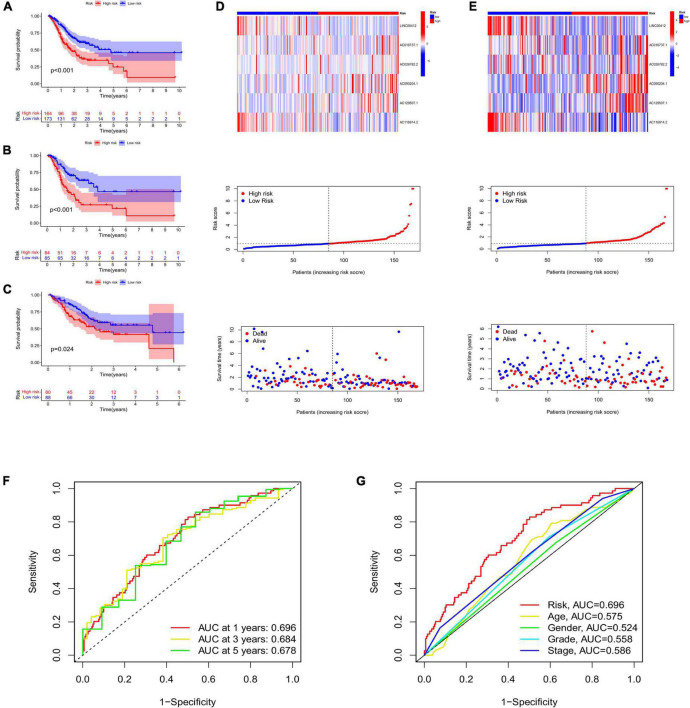
**(A)** Survival curve of low- and high-risk groups in all samples. The survival analysis of low- and high-risk groups in the training group **(B)** and the test group **(C)**. Heatmap displaying the expression of risk lncRNAs between low- and high-risk groups, and the ranked dot plot showing the risk score distribution in the training group **(D)** and the test group **(E)**. **(F)** The AUC values of 1-, 3-, and 5-year survival. **(G)** Comparison of AUC between risk signature and age, gender, grade, and stage. AUC, area under the curve.

**FIGURE 4:**
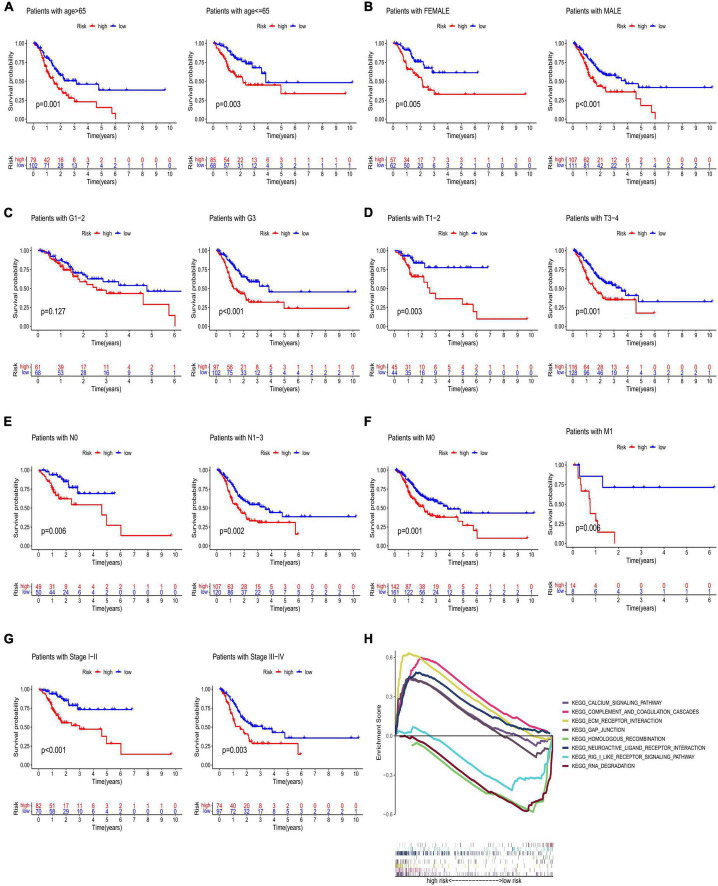
Kaplan–Meier analyses of age **(A)**, gender **(B)**, grade **(C)**, T stage **(D)**, N stage **(E)**, M stage **(F)**, and stage **(G)**. **(H)** GSEA of low- and high-risk groups. GSEA, gene set enrichment analysis.

High-risk lncRNAs included AC016737.1, AC026782.2, AC090204.1, and AC129507.1. Low-risk lncRNAs included LINC00412 and AC116914.2 (Figure 2E). We found that LINC00412, AC016737.1, AC026782.2, and AC090204.1 were highly expressed in CRC. AC129507.1 was downregulated in CRC (Figure 2C). The relationship between CRGs and the lncRNAs is displayed in Figure 2D. The low-risk group showed better survival outcomes than the high-risk group (Figures 2E, 3A). The training group and test group confirmed this conclusion (Figures 3B–E). The area under the curve (AUC) values demonstrated that our prognostic signature had moderate performance (Figure 3F). The AUC values of age, gender, grade, and tumor stage were 0.575, 0.524, 0.558, and 0.586, respectively, indicating that the risk model had the best predictive ability (Figure 3G). The Kaplan-Meier survival curve further proved that this risk signature applied to patients of any age, gender, grade, and TNM stage (Figures 4A–G).

### Gene set enrichment analysis and nomogram construction

**FIGURE 5:**
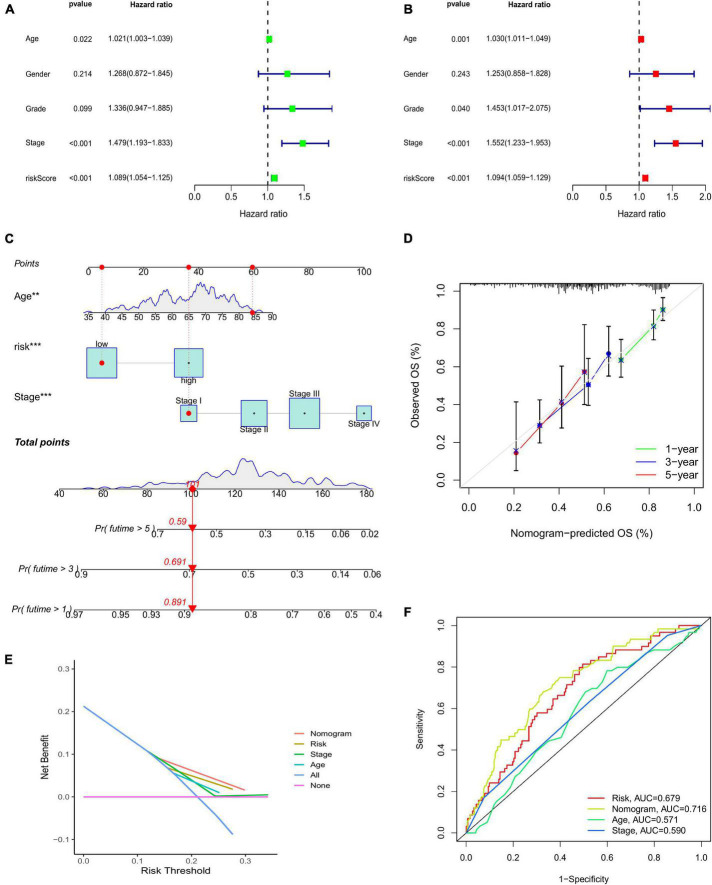
**(A)** Univariate Cox regression analysis. **(B)** Multivariate regression analysis. **(C)** Nomogram to predict the 1-, 3-, and 5-year survival probability for patients with CRC. **(D)** The calibration curves of 1-, 3-, and 5-year survival. **(E)** Decision curve analysis comparing stage, age, risk score, and the nomogram. **(F)** AUC values of the stage, age, risk score, and nomogram. CRC, colorectal cancer; AUC, area under the curve.

The results of the GSEA showed that the main functional pathways in the high-risk group were the calcium signaling pathway, GAP junction, extracellular matrix receptor interaction, and complement and coagulation cascades. The main functional pathways in the low-risk group were homologous recombination, neuroactive ligand-receptor interaction, retinoic acid-inducible gene-I-like receptor signaling pathway, and RNA degradation (Figure 4H). The univariate regression analysis showed that age (hazard ratio [HR]: 1.003–1.039; *P* < 0.05), stage (HR: 1.193–1.833; *P* < 0.001), and risk score (HR: 1.054–1.125; *P* < 0.001) were associated with prognosis (Figure 5A). It was demonstrated that age (HR: 1.011–1.049; *P* < 0.001), stage (HR: 1.233–2.075; *P* < 0.001), and risk score (HR: 1.059–1.129; *P* < 0.001) were found to be independent prognostic factors (Figure 5B). A prognostic nomogram was also developed for the prediction of the survival time (Figure 5C). The calibration curves for the 1-, 3-, and 5-year survival rates confirmed the accuracy of the nomogram (Figure 5D). The decision curve analysis indicated that the nomogram had a better predictive ability for survival time than the stage, age, and risk score (Figure 5E). The AUC values of the stage, age, risk score, and nomogram were 0.590, 0.571, 0.679, and 0.716, respectively (Figure 5F).

### Analyses of immune infiltration, immune checkpoints, and clinical data

**FIGURE 6:**
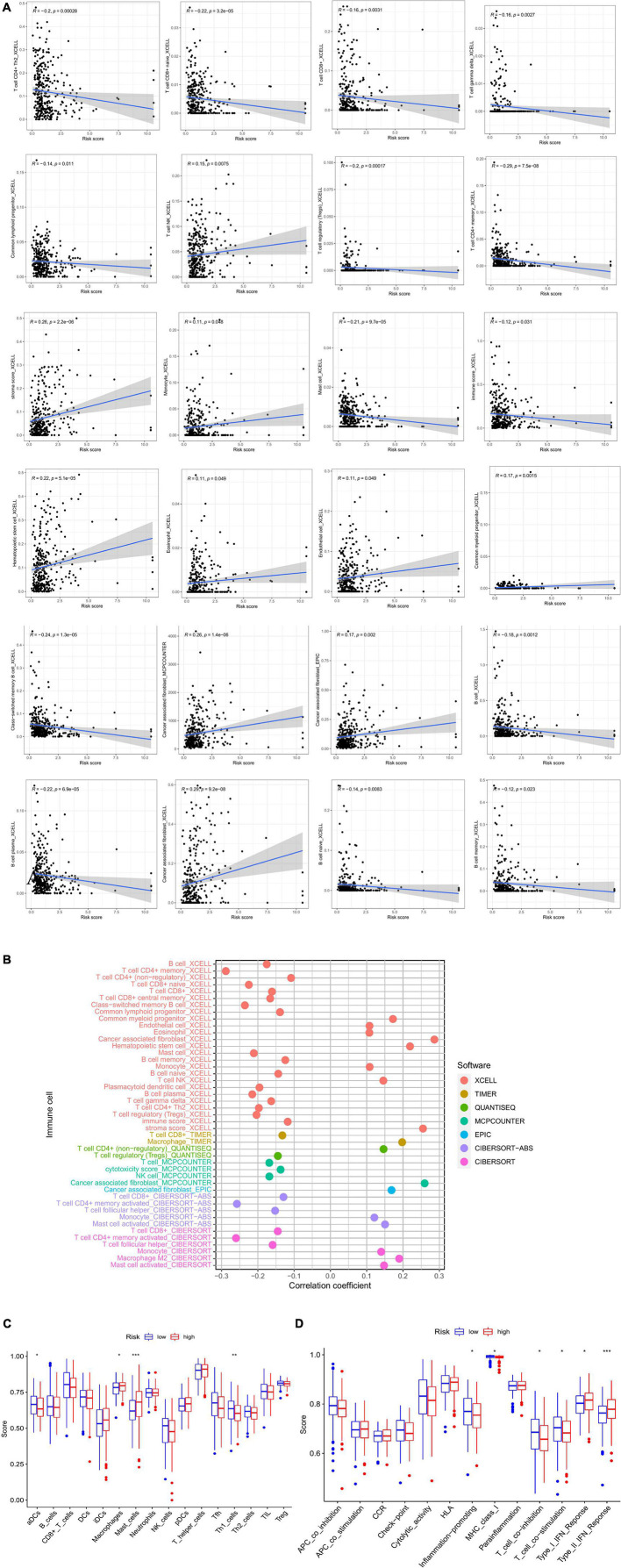
**(A)** Correlation between risk score and immune cells. **(B)** XCELL, TIMER, QUANTISEQ, MCPCOUNTER, EPIC, and CIBERSORT to analyze the immune landscape of patients with CRC. **(C,D)** ssGSEA analysis. CRC, colorectal cancer; GSEA, gene set enrichment analysis.

**FIGURE 7:**
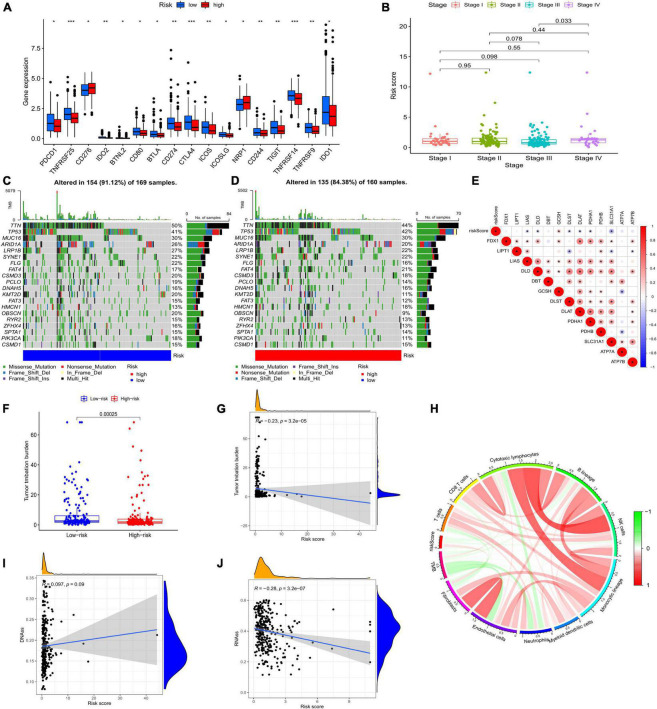
**(A)** Expression of immune checkpoints in the two risk groups. **(B)** Clinical features of the model. Somatic mutations in the low-risk group **(C)** and the high-risk group **(D)**. **(E)** Association between risk score and CRGs. **(F)** TMB of the two risk groups. **(G)** The relationship between TMB and risk score. **(I,J)** Correlation between CSC and risk score. **(H)** Circle picture displaying the relationship between TMB and immune cells. CRGs, cuprotosis-related genes; TMB, tumor mutational burden; CSC, cancer stem cell;

The low-risk group had more immune cell infiltration (Figure 6B). Additionally, we discovered that some immune cell types had positive correlations with risk scores while others had negative correlations (Figure 6A). The results of ssGSEA indicated that some activities, such as inflammation promotion, were upregulated in the high-risk group (Figures 6C–D). There was a differential expression of 17 immune checkpoints, of which 16 (94.12%) had higher expression levels in the low-risk group (Figure 7A). Meanwhile, higher risk scores were observed in late-stage CRC (Figure 7B).

### Characteristics of tumor mutational burden, cancer stem cells, and microsatellite instability

**FIGURE 8:**
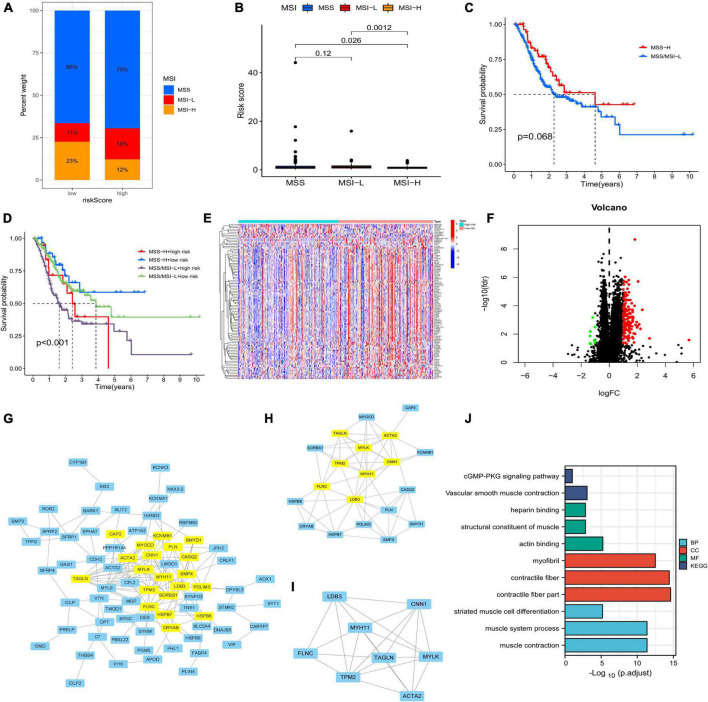
**(A,B)** Relationship between MSI and risk score. **(C)** Correlation between survival time and MSS/MSI-L and MSI-H. **(D)** Survival analysis integrating MSI status and risk signature. **(E)** Differentially expressed genes (DEGs) between the two risk groups. **(F)** Volcano plot of DEGs. **(G–I)** Core DEGs in the two risk groups. **(J)** Gene ontology and Kyoto Encyclopedia of Genes and Genomes analyses of DEGs. MSI, microsatellite instability.

Both low- and high-risk groups had the same top five mutated genes. However, the low-risk group demonstrated a higher mutation probability (Figures 7C–D). TMB was high in the low-risk group (Figure 7F). TMB and risk score were inversely correlated (Figure 7G). Differences in TMB between the two groups may be related to endothelial cells and neutrophils (Figure 7H). Patients with high-risk scores had lower RNA and higher DNA markers in CSCs than patients with low-risk scores (Figures 7I–J). Figure 7E illustrates the relationship between risk scores and CRGs. MSS/MSI-L was strongly associated with higher risk scores (Figures 8A–B). There was no association between survival rates and MSS/MSI-L and MSI-H status. However, the MSI-H + low-risk score had the most favorable prognosis compared with the MSS/MSI-L + high-risk score, the MSS/MSI-L + low-risk score, and the MSI-H + high-risk score groups (Figure 8D).

### Analyses of chemotherapeutic drug sensitivity and differential genes

**FIGURE 9:**
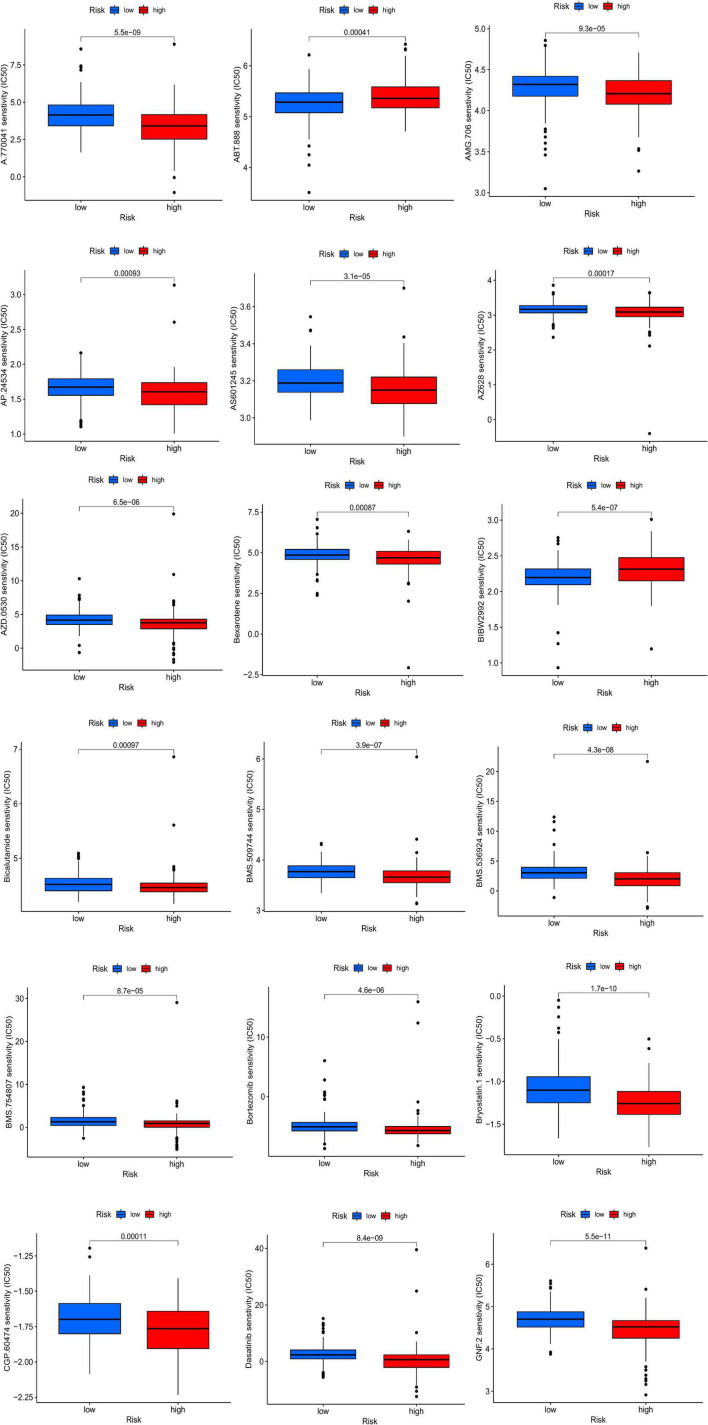
Drug sensitivity analysis between high- and low-risk groups.

After running the limma package, we selected 140 DEGs (Figure 8E), including 131 overexpressed and 9 underexpressed genes (Figure 8F). Based on the scores of Betweenness, Closeness, Degree, Eigenvector, LAC, and Network, we performed two screenings and obtained eight core genes (Figures 8G–I). The Gene Ontology and Kyoto Encyclopedia Genes and Genomes analyses based on the 140 DEGs showed that the cGMP-PKG signaling pathway, heparin binding, and contractile fiber may be the main biological functions. Finally, we screened a total of 37 chemotherapeutic drugs to evaluate the differences in sensitivity between the two groups. Out of these 37 chemotherapeutic drugs, 32 (86.49%) had higher IC_50_ values in the low-risk group than in the high-risk group. This suggests that high-risk patients may be more sensitive to chemotherapy drugs, contributing to a more favorable prognosis (Figure 9).

## Discussion

Numerous studies have demonstrated that cell death is related to tumor occurrence and progression (16). The known mechanisms of cell death mainly include ferroptosis, pyroptosis, necroptosis, apoptosis, and autophagy (17–21). Tsvetkov et al. first described cuprotosis, a new mechanism of cell death (12). However, the relationship between cuprotosis and CRC is unclear, especially CRLNCs. In this study, we identified CRLNCs using bioinformatics studies. Six lncRNAs (i.e., LINC00412, AC016737.1, AC026782.2, AC090204.1, AC129507.1, and AC116914.2) were then used to construct a prognostic model for survival time prediction, immune infiltration, and chemotherapy drug sensitivity of CRC. LINC00412 was included in the 10 biomarkers for the construction of the cardia cancer prognostic model and contributed to the modification of the prognostic models by Xin et al. (22). Taniguchi-Ponciano et al. found that LINC00412 was upregulated in all kinds of pituitary tumors (23). Zhang et al. provided evidence that AC016737.1 was associated with the inflammatory response of CRC, and this association was later validated by constructing a prognostic model (24). Chen et al. used hypoxia-related lncRNAs, which included AC016737.1, to construct a model for predicting the outcome of CRC. AC016737.1 was also used in the m6A-modified lncRNA prognostic nomogram by Song et al. and the immune-related lncRNA pair model by Shenglei et al. for CRC prognosis. Zha et al. discovered that AC129507.1 played a role in predicting prognosis and multiple tumor-related pathways in CRC (25). AC129507.1 was also used in the exosome-related lncRNA CRC prognostic model by Li et al. as well as in the survival prediction model of gastroesophageal junction adenocarcinoma by Song et al. AC116914.2 was involved in autophagy, m^6^A RNA methylation, and hypoxia in head and neck squamous cell carcinoma. Meanwhile, AC116914.2 was associated with survival and immune cell infiltration (26–28). The results of q-RT PCR showed that AC016737.1, AC026782.2, AC090204.1, and AC129507.1 were highly expressed in tumor cells compared with normal cell, while LINC00412 and AC116914.2 were low expressed using Tukey’s HSD posttest as the method of multiple comparisons. This is consistent with the fact that AC016737.1, AC026782.2, AC090204.1, and AC129507.1 were high-risk genes, and LINC00412 and AC116914.2 were low-risk genes.

The tumor microenvironment is well known to be the site of tumor survival, with multiple components interacting to form a complex and polymorphic environment (29). Immune cell infiltration is one of the key components of TME. Comprehensive analysis of immunological signatures in the TME could facilitate the progress of native and effective immunotherapeutic strategies, as well as the discovery of highly effective biomarkers (30). TME also plays an important role in regulating tumor sensitivity to treatment (31). B cell is the most crucial humoral immune cell, mediating the antitumor response. It is associated with a favorable prognosis and immunotherapy response (32). Notably, the low-risk group had a higher infiltration level of B cell, B cell memory, B cell plasma, and naïve B cell. CD4 + T cells can kill tumors either directly by destroying the tumor cells or indirectly by mediating TME regulation. In addition, CD4 + T cells also can promote gene expression and differentiation of CD8 + T cells (33–35). As a result, we found that some T cell types, including T cell CD4 + memory and T cell gamma delta, were present in higher levels in the low-risk group than in the high-risk school. The first line of defense in identifying tumors is the ability of CD8 + T cells to recognize MHC class I molecules expressed by tumor cells. CD8 + T cells are the most efficient immune cells against cancer (36). In this study, the infiltration level of CD8 + T cells and CD8 + central memory T cells were higher in the low-risk group than in the high-risk group. Cancer-associated fibroblasts (CAFs) in the TME have been shown to promote the proliferation of multiple tumors by secreting a variety of biological factors to suppress the immune response (37). Various molecules, such as epidermal growth factor and interleukin-6, can be secreted by CAFs to enhance cell proliferation, tumor invasion and metastasis, and epithelial-mesenchymal transition. Notably, a higher infiltration level of CAFs was observed in the high-risk group than in the low-risk group, possibly resulting in the difference in prognosis between the two groups. Meanwhile, we also discovered that the patients with low-risk scores obtained higher immune scores and lower stromal scores than those with high-risk scores, which further gives a reasonable explanation for the difference in prognosis between the two groups.

Immune checkpoint inhibitors have become a promising treatment strategy in almost all kinds of malignant tumors. Several clinical trials involving nivolumab, pembrolizumab, ipilimumab, avelumab, and durvalumab have either been completed or are currently being conducted. In the low-risk group, we found the overexpression of 16 immune checkpoints, which could reveal potential immune therapy targets and help develop combination therapies and predictive biomarkers.

Cancer genomics studies have found that most cancers develop with the accumulation of somatic gene mutations (38). In this study, a higher mutation probability was detected in the low-risk group than in the high-risk group. It is widely known that TMB and MSI are predictive biomarkers of immunotherapy response. High TMB and MSI-H appear to be associated with favorable immunotherapy response and prognosis (39, 40). Our findings also confirmed this conclusion and may contribute to revealing potential therapeutic targets.

We also found that patients in the low-risk group were more sensitive to chemotherapy than patients in the high-risk group. Fluorouracil-based adjuvant chemotherapy is recommended for resected stage III and some stage II colon cancers to improve patient survival. Several studies have concentrated on the addition of oxaliplatin to fluorouracil as a novel standardized CRC chemotherapy (41–44). The standard course of adjuvant chemotherapy is 6 months. A major disadvantage of oxaliplatin chemotherapy is cumulative sensory neuropathy. In a clinical trial, 3-month adjuvant chemotherapy in low-risk stage III (not T4 or N2) colon cancer did not compromise treatment efficacy but reduced drug toxicity (such as neuropathy) (45). Chemotherapy sensitivity is vital for CRC treatment (46). Our prognostic signature can help make chemotherapy more effective or tailor treatment to each individual, which is critical for survival.

Our study also had several limitations. Comprehensive and detailed *in vitro* and *in vivo* experiments are still needed to further validate our conclusion. Also, more clinical samples need to be included.

## Conclusion

Based on CRLNCs, a prognostic signature was constructed to predict the survival and chemotherapy sensitivity of patients with CRC. In summary, our study analyzed the role of CRLNCs in CRC and provided new targets and strategies for CRC therapy.

## Data availability statement

The original contributions presented in the study are publicly available. This data can be found here: https://portal.gdc.cancer.gov/repository.

## Ethics statement

The studies involving human participants were reviewed and approved by Ethics Committee of the Second Hospital of Jilin University. Written informed consent for participation was not required for this study in accordance with the national legislation and the institutional requirements.

## Author contributions

TL: work concept or design. JZ: data collection. WL and HD: draft the manuscript. GY: make important revisions to the manuscript and approved the final manuscript. All authors read and approved the final manuscript.
