# Supplementary material for: A prognostic signature based on cuprotosis-related long non-coding RNAs predicts the prognosis and sensitivity to chemotherapy in patients with colorectal cancer
Source: Front Med (Lausanne). 2022 Nov 16;9:1055785. doi: 10.3389/fmed.2022.1055785 (PMC9709405; doi:10.3389/fmed.2022.1055785)
Supplement: Supplementary file 2 [file Data_Sheet_1.docx]

**Supplementary material**

**Construction and validation of a prognostic lncRNA signature**

First, we equally divided all tumor samples into two groups, the train group and the test group. The train group was applied to establish a risk signature, and the test group was utilized to validate the repeatability of the signature. Then, the univariate regression analysis was developed to select DELNCs linked with prognosis. LASSO regression analysis for prognostic-related DELNCs using the glmnet package in R was conducted to prevent overfitting. And multivariate regression analysis were carried out to generate a risk score formula with a prognostic signature. In terms of the formula, each patient in the train group obtained a risk score. A median risk score was calculated according to total scores in the train group. In the process of regrouping, the median will be used as the dividing line, and those with a score higher than the median will be assigned to the high-risk group. Similarly, those with a score lower than the median will be classified as a low-risk group. We also divided the samples of the test group into two risk groups. The comparison of the time of survival between the two groups was completed with the Kaplan-Meier analysis.Dot plots were drawn to observe changes in risk score versus survival status and overall survival. We construct the ROC curves and obtain the area under the ROC curve (AUC) for 1, 3, and 5-year OS using the survival ROC package. Meanwhile, we also compared the accuracy of age, gender, grade, stage, and the risk signature in the prediction of the 1-year survival time of patients. Further verifying the feasibility of the model was demonstrated by the survival analyses of age, gender, grade, TNM, and stage.

**GSEA analysis and a nomogram**

We explored the functional pathways in the two risk groups through the GSEA software. The filter criterion according to the c2.cp.kegg.v7.5.1symbols.gmt gene set was |normalized enrichment score (NES)| > 1.5, and nominal (NOM) p-value < 0.05. The univariate and multivariate regression analyses were utilized to select independent prognostic factors in patients with GC, including age, stage, and risk score. Then, to improve the clinical application of the model, we also developed a nomogram to predict the 1, 3, and 5 -year survival probability for GC patients. The calibration curves of 1, 3, and 5 -year were assessed the accuracy of the nomogram. At the same time, AUC and Decision Curve Analysis (DCA) were applied to compare stage, age, risk score, and the nomogram in the predictive ability for survival time.

**Analyses of immune landscape, immune checkpoints and clinical data**

To comprehensively analyze the infiltration of immune cells, we analyzed the immune infiltration of GC patients via various methods, including XCELL, TIMER, QUANTISEQ, MCPCOUNTER, EPIC, CIBERSORT, and ssGSEA. Various methodes, such as XCELL, TIMER, QUANTISEQ, MCPCOUNTER, EPIC, CIBERSORT, and ssGSEA, were used to analyze immune infiltration of GC patients. Expression of 48 immune checkpoints, such as LAG3, CTLA4, CD80, PDCD1, HHLA2, VTCN1, and so on, was compared between in the two risk groups using the limma package. Subsequently, we analyzed clinicopathological features between the groups of high-risk and low-risk.
