# Supplementary figures and images for: A prognostic signature based on cuprotosis-related long non-coding RNAs predicts the prognosis and sensitivity to chemotherapy in patients with colorectal cancer
Source: Front Med (Lausanne). 2022 Nov 16;9:1055785. doi: 10.3389/fmed.2022.1055785 (PMC9709405; doi:10.3389/fmed.2022.1055785)

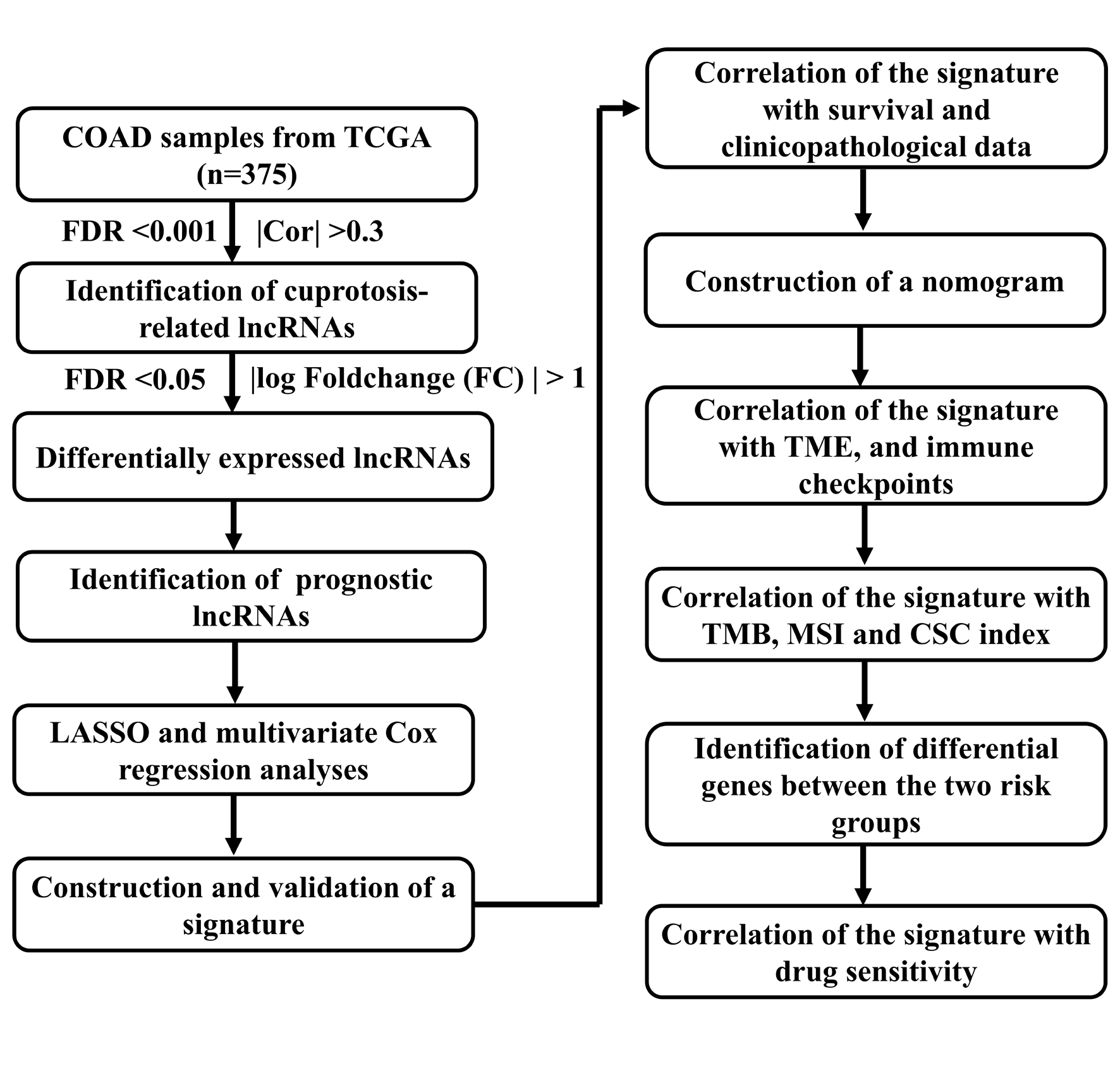

Supplement: Supplementary file 3 [file Image_1.TIF]

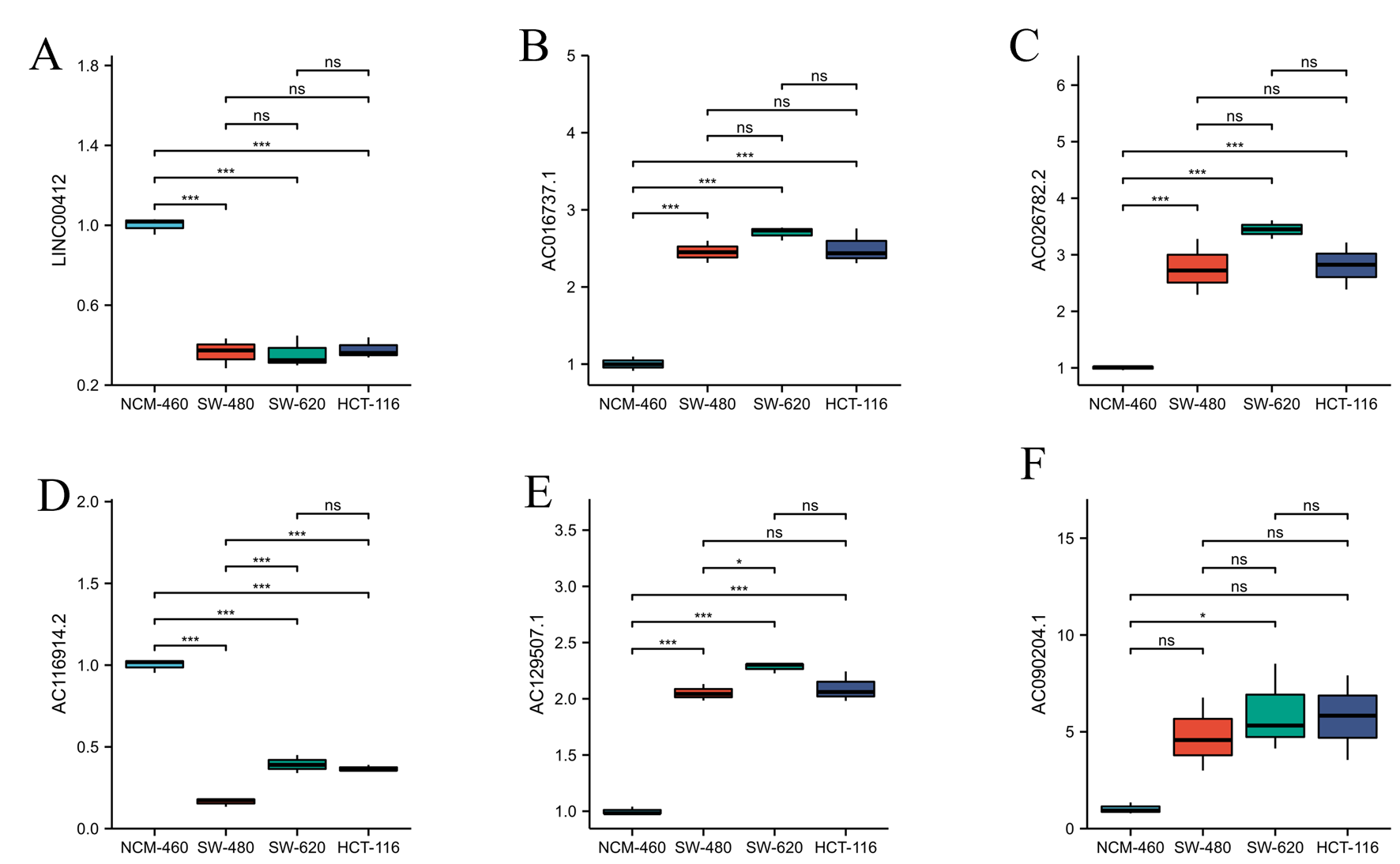

Supplement: Supplementary file 4 [file Image_2.TIF]

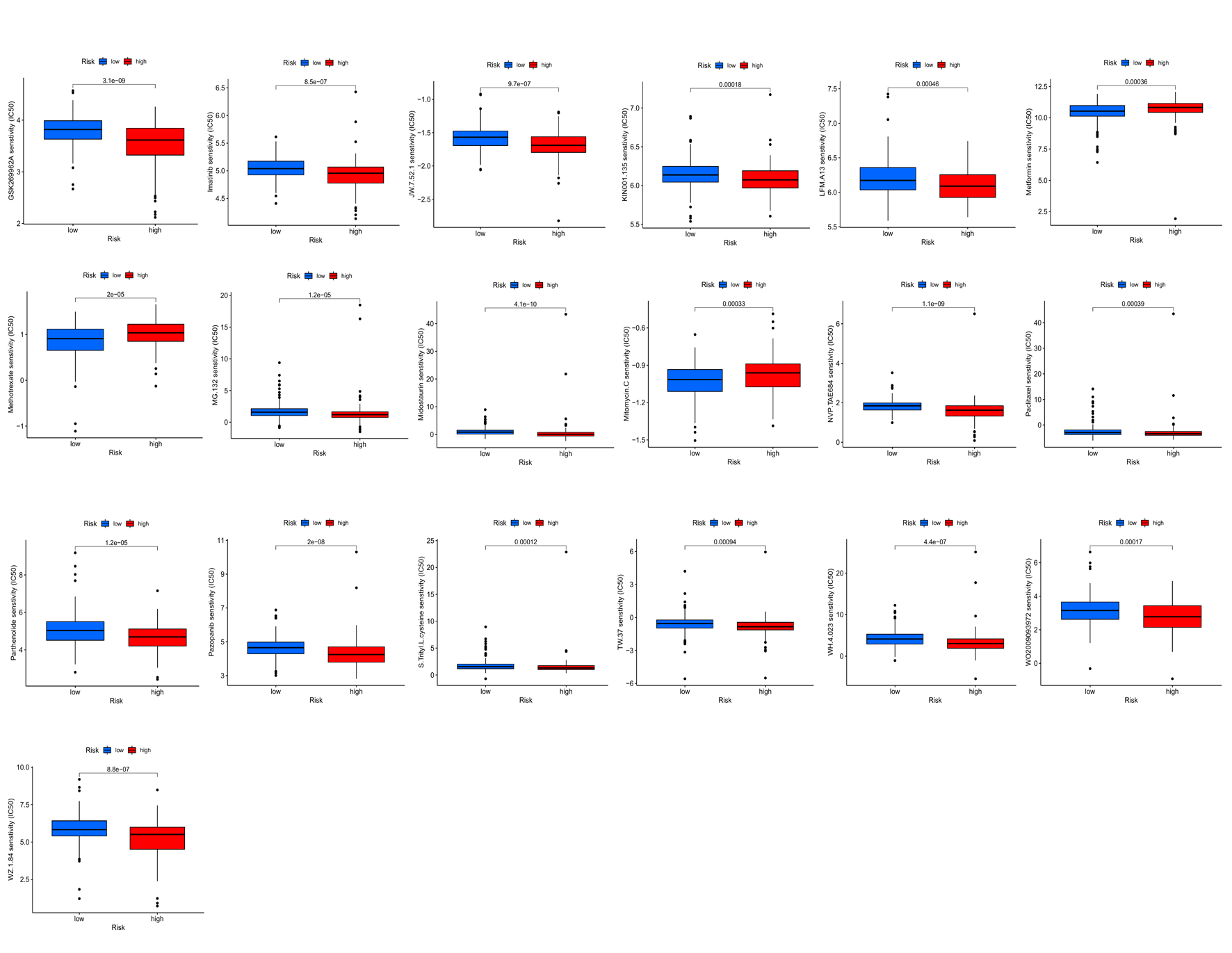

Supplement: Supplementary file 5 [file Image_3.TIF]
